# Should autism spectrum disorder be considered part of CHARGE syndrome? A cross-sectional study of 46 patients

**DOI:** 10.1186/s13023-020-01421-9

**Published:** 2020-06-03

**Authors:** Véronique Abadie, Priscilla Hamiaux, Stéphanie Ragot, Marine Legendre, Gaelle Malecot, Alexia Burtin, Tania Attie-Bitach, Stanislas Lyonnet, Frédéric Bilan, Brigitte Gilbert-Dussardier, Laurence Vaivre-Douret

**Affiliations:** 1 Faculty of Medicine, University of Paris, Paris, France; 2 Department of Paediatrics, Necker-Enfants Malades University Hospital, AP-HP, Paris, France; 3 INSERM Unit 1178/1018-CESP, University of Paris Sud-Paris Saclay, UVSQ Villejuif and Paris Descartes, Sorbonne Paris Cité, Paris, France; 4 Institute Imagine, Paris, France; 5 Service de Pédiatrie Générale, Hôpital Universitaire Necker, 149 rue de Sèvres, 75015 Paris, France; 6 INSERM, CIC 1402, Poitiers, France; 7 Genetics Unit, CHU de Poitiers, Poitiers, France; 8 University of Poitiers, Faculty of Medicine and Pharmacy, Poitiers, France; 9 Genetics Unit, University of Bordeaux, Bordeaux, France; 10 Genetics Unit, University Hospital of Nancy, Nancy, France; 11 Department of Genetics, Necker University Hospital, APHP, Paris, France; 12 EA 3808, University of Poitiers, Poitiers, France; 13 Department of Child Psychiatry, Necker University Hospital, AP-HP, Paris, France; 14 University Institute of France (IUF), Paris, France

**Keywords:** CHARGE syndrome, CHD7, Behavioral disorders, Behavior, Sensory deficits, Autistic traits, Autism, Autism spectrum disorder

## Abstract

**Background:**

Behavioral problems are an important issue for people with CHARGE syndrome. The similarity of their behavioral traits with those of people with autism raises questions. In a large national cross-sectional study, we used specific standardized tools for diagnosing autism (Autism Diagnostic Interview-Revised and Diagnostic and Statistical Manual of Mental Disorders, 5th edition, DSM-5) and evaluating behavioral disorders (Developmental Behavior Checklist-Parents, DBC-P) to investigate a series of individuals with CHARGE syndrome, defined by Verloes’s criteria. We evaluated their adaptive functioning level and sensory particularities and extracted several data items from medical files to assess as potential risk factors for autism and/or behavioral disorders.

**Results:**

We investigated 64 individuals with CHARGE syndrome (35 females; mean age 10.7 years, SD 7.1 years). Among 46 participants with complete results for the Autism Diagnostic Interview-Revised (ADI-R), 13 (28%) had a diagnosis of autism according to the ADI-R, and 25 (54%) had a diagnosis of autism spectrum disorder (ASD) according to the DSM-5 criteria. The frequency of autistic traits in the entire group was a continuum. We did not identify any risk factor for ASD but found a negative correlation between the ADI-R score and adaptive functioning level. Among 48 participants with data for the DBC-P, 26 (55%) had behavioral disorders, which were more frequent in patients with radiological brain anomalies, impaired adaptive functioning, later independent walking, and more sensory particularities.

**Conclusions:**

ASD should be considered to be an independent risk requiring early screening and management in children born with CHARGE syndrome.

## Introduction

CHARGE syndrome (CS) is a rare genetic condition that can feature multiple disabilities, including variable occurrence of (C) coloboma, (H) heart defects, (A) atresia of choanae, (R) retardation of growth or development, (G) genital hypoplasia, and (E) ear abnormalities and deafness [1]. Other possible malformations and deficits added later include arhinencephaly resulting in hyposmia, anomalies of the semicircular canals producing vestibular dysfunction, and cranial nerve and brainstem dysfunction, which lead to feeding and respiratory difficulties during the first years of life [2–4]. Most individuals (around 80%) with CS have mutations in the chromodomain-helicase-DNA-binding protein 7 *(CHD7)* gene, but the diagnosis of CS remains clinical. Diagnostic criteria were proposed by K.D. Blake in 1998 and were revised in 2006 and 2007 by A. Verloes et al. [5–9]. More recently, Hale et al. proposed inclusion of the pathogenic *CHD7* variant status as a major criterion of CS diagnosis [10].

Behavioral disorders are neither specified nor included in the syndrome definition. However, for many years, parents, caregivers and professionals have reported that children with CS often have behavioral disorders [11–14], anxiety [11], obsessive-compulsive disorders [12, 15], and sensory particularities [11, 15, 16]. No consensual definition exists for behavioral disorders, which can nonetheless be described as behaviors that deviate from social, cultural, or developmental norms and significantly harm the individual or his/her environment [17]. In CS, some of these behaviors are similar to autistic traits, which explains why authors talk about “autistic-like behaviors” when describing them [18]. Individuals with CS appear to have better communication skills and more interest in social relationships than people with autism [19–22]. Compared with deaf-blind people, individuals with CS show fewer self-regulation abilities along with more ritualistic, stereotyped, and self-stimulation behaviors [18]. The frequency of autism spectrum disorder (ASD) in individuals with CS ranges from 9 to 68% depending on the ASD definition used [19]. In any case, few studies have investigated individuals with CS with standardized tools specific for the diagnosis of autism or ASD.

Our study had two objectives: first to identify ASD and behavioral disorders in a large series of patients with CS, by using specific, standardized tools, and second, to search for determinants of these challenging behaviors. We analyzed eight potential determinants: global somatic severity, medical severity during the first year of life, radiological brain anomalies, sensory deficits (visual and auditory), age, adaptive functioning level, and sensory particularities. Finally, we discuss the advantages and disadvantages of including ASD as an independent clinical feature of CS.

## Patients and methods

### Patients

This study is one component of a cross-sectional national study that analyzed the phenotypes and genotypes of 117 individuals with CS. Clinical diagnostic criteria were selected from Sanlaville & Verloes [8, 9] (Table 1). This portion of the study included only participants living in the Paris and Nancy regions (whose parents agreed to participate), because psychologists (PH and AB) were available only in these regions to perform it. All parents were informed and gave their written consent for their child’s participation. All assessments took place on site during a 2-day period and were completed by several telephone calls. We extracted the clinical data from the dataset for the clinical part of the national study. This work was supported by the French Ministry of Health and approved by the national ethics committee (no. ID-RCB: 2010-A00700–39).

**Table 1:** Diagnostic criteria for CHARGE syndrome by Verloes (2005), updated by Blake (2006) and Sanlaville (2007)

**Major criteria**	Coloboma
	Choanal atresia and/or cleft lip and palate
	Semicircular canals agenesis/hypoplasia
	Arhinencephaly and/or anosmia
**Minor criteria**	Cranial nerves VII to XII palsy
	Hypothalamo-hypophyseal dysfunction
	External or middle ear anomalies
	Intellectual disabilities
**Typical CHARGE syndrome**	3 major or 2 major + 2 minor criteria
**Partial CHARGE syndrome**	2 major + 1 minor criteria
**Atypical CHARGE syndrome**	2 major + 0 minor or 1 major + 3 minor criteria

### Methods

To investigate ASD, we interviewed parents with the Autism Diagnostic Interview Revised (ADI-R) questionnaire and used the responses to the ADI-R to determine the presence of the criteria described in the Diagnostic and Statistical Manual of Mental Disorders, 5th Edition (DSM-5) [23–25].

### ADI-R

The ADI-R is a clinical semi-structured parental interview investigating autistic behaviors, for children from 2 years of age to adulthood. We chose the ADI-R because it is an appropriate tool for diagnosing ASD in individuals with various intellectual abilities. We used the algorithm that scores a critical number of questions and the version validated in French [26]. This questionnaire explores three domains: 1) social interaction, 2) communication, and 3) restricted, repetitive, and stereotyped behaviors, with a total score for each. Each domain has a cutoff score above which ASD is suspected. If the cutoff scores are reached for all three domains, the autism diagnosis is confirmed. When children are 5 years or younger, parents answer each question about current symptoms. When the patient is older than 5 years, the parents answer twice, once for “currently” and once for when the child was about 5 years old (hereafter “at age 5”), when the symptoms are usually most evident.

Each domain contains subdomains, each with several items. The social interaction domain contains four subdomains: failure to use nonverbal skills (gestures, posture, gaze, and expression) to regulate social interactions, failure to develop peer relationships, lack of seeking to share pleasure, and lack of social-emotional reciprocity. The communication domain contains two subdomains for all children — delay in oral language not compensated by gesture, and lack of varied spontaneous make-believe play — and two more only for children with oral language skills: relative failure to initiate or sustain conversational exchange, and stereotyped, repetitive, and idiosyncratic language. The restricted, repetitive, and stereotyped behaviors domain contains four subdomains: encompassing preoccupations or restricted interests, compulsive adherence to rituals, stereotyped or repetitive motor mannerisms, and preoccupation with part-objects and non-functional elements of materials.

Each score ranges from 0 (absence or very little presence of the abnormal behavior) to 2 (strong presence of the behavior). The cutoff is 10 for the social interaction domain; 7 and 8 for the communication domain for nonverbal and verbal participants, respectively; and 3 for the restricted, repetitive, and stereotyped behaviors domain.

To compare the profiles of the patients of our series with those of people with classical autism or with intellectual disability, we used the ADI-R results of 25 children with autism and 23 defined as “mentally handicapped-language impaired”, previously published (means and standard deviations [SD] in the ADI-R manual (Western Psychological Services) [23, 24].

### DSM-5

We searched for ASD according to DSM-5 criteria (25) by using a posteriori the ADI-R answers from parents for their children at age 5 (or current for children aged less than 5). The diagnosis of ASD in DSM-5 involves two main criteria: A: Persistent deficits in social communication and social interaction across multiple contexts, and B: Restricted, repetitive patterns of behavior, interests, or activities. Domain A includes 3 criteria of symptoms: 1) deficits in social-emotional reciprocity, 2) deficits in nonverbal communicative behaviors used for social interaction, et 3) deficits in developing, maintaining, and understanding relationships. Domaine B includes 4 criteria: 1) stereotyped or repetitive motor movements, use of objects, or speech, 2) insistence on sameness, inflexible adherence to routines, or ritualized patterns or verbal nonverbal behavior, 3) highly restricted, fixated interests that are abnormal in intensity or focus, and 4) hyper- or hyporeactivity to sensory input or unusual interests in sensory aspects of the environment. According to DSM-5, the diagnosis of ASD is made if a person manifests 3 of 3 criteria in domain A and 2 of 4 in domain B. Moreover, DSM-5 classifies symptoms by three severity levels: level 1, requiring support; level 2, requiring substantial support; and level 3, requiring very substantial support (25).

### Developmental behavior checklist–parents (DBC-P)

To differentiate autism from behavioral disorders, we used a specific scale to assess behavior problems, the DBC-P, a parental questionnaire that includes 96 items evaluating disturbed emotions and behaviors in children aged from 4 to 18 years, with intellectual disabilities ranging from mild to profound [27]. We chose the DBC-P because it evaluates behavior disorders in people with any level of intellectual disability. It has been used in two other studies of individuals with CS [13, 28]. The parents evaluate the presence (or absence) and the frequency (or intensity) of their child’s abnormal behavior during the past 6 months (0, never; 1, sometimes; 2, often). Results are expressed as raw scores and then as percentiles of the total sample. This questionnaire makes it possible to calculate a total score (total behavioral disorders), with a cutoff of 46 (weighted score = 0.484). The DBC-P contains 5 subscales describing various psychopathological areas: disruptive/antisocial (externalizing behavior disorders), self-absorbed, communication disturbances, anxiety (internalizing behavior troubles), social relationships, and a few items for autism screening. We calculated the weighted means and SDs for each subscale for comparison, although the number of items varies between the subscales.

### Vineland adaptive behavior scale (VABS-II)

To analyze adaptive functioning, we used the VABS-II, a semi-structured interview with the parent/caregiver to investigate communication, daily living skills (autonomy), socialization, and motor skills. Because the VABS-II involves an indirect assessment, it can be used even for individuals with severe intellectual impairment. For various behaviors and skills, the parent/caregiver is asked if the individual does not do it, sometimes does it, or often does it (mean = 100, SD = 15 points). It can be used regardless of the subject’s age. The VABS-II has been translated into French and validated in the French population [29–31].

### Dunn’s sensory profile

Dunn’s sensory profile investigates specific behaviors related to sensory particularities. It is a parental questionnaire widely used for investigating sensory dysfunctions. It includes 125 items divided into three main sections: sensory processing (divided into six subsections corresponding to modalities), sensory modulation (divided into combinations of input), and behavioral and emotional responses (divided into three subsections). The parent is asked about the frequency of various behaviors. The higher the score, the greater the sensory particularities. Dunn’s questionnaire has been validated in a French population [32, 33].

### Global somatic severity

We calculated a score, previously used by Hartshorne et al. [12, 18], to estimate the global somatic severity of CS by attributing 1 point for the presence of each one of its 13 main impairments or malformations: malformation of semicircular canals or inner ear, frequent ear infections, eye coloboma, cardiac malformation, skeleton or spine anomaly, genital anomaly or delayed puberty, growth retardation, facial palsy, renal or urinary tract malformation, choanal atresia, cleft lip and/or palate, esophageal atresia, and microcephaly. The total score ranges from 0 (mild) to 13 (most severe syndrome).

### Medical severity during the first year of life

Because the severity of the infant’s medical condition, especially during the first months of life, is suspected to generate stress for parents and babies, we created a score to take it into account by attributing 1 point for the presence of each of the following four events during the first year of life: tracheotomy, tube feeding for more than 6 months, more than 6 months spent at the hospital, and at least one surgical intervention. The total medical severity score during the first year of life ranges from 0 to 4.

### Radiological brain anomalies

We used brain MRI results from the participants’ medical charts to create a brain anomaly score, calculated as 1 = no brain anomaly (except olfactory bulbs and/or semicircular canal anomalies); 2 = asymmetric ventricular dilatation and/or simple cortical atrophy; 3 = anomalies of the cerebellum and/or corpus callosum; or 4 = gyration anomaly or other severe brain malformations.

### Visual impairment

We created a visual impairment score for visual acuity once corrected, if available, and the location of the retinal coloboma. This score was 0 for individuals with either no coloboma or good visual acuity (> 6/20 for both eyes); 1, visual acuity is 2/20 to 6/20 or there is a unilateral retinal coloboma that does not affect the macula or a bilateral coloboma that is only peripheral; or 2, visual acuity is < 2/20 or bilateral coloboma affects both maculae.

### Hearing loss

We created a hearing loss score according to the severity of hearing loss (without hearing aids), scored as 0, hearing loss < 20 dB or no hearing loss; 1, hearing loss 20 to 40 dB (mild hearing loss); 2, hearing loss 41 to 70 dB (moderate); 3, hearing loss 71 to 90 dB (severe); or 4, hearing loss > 90 dB (profound).

### Statistical analysis

Quantitative data are described with means (SD) and categorical data with frequencies (%). We compared the mean ADI-R scores for all participants with those in the ADI manual with classical autism and with intellectual disability (Wilcoxon test). To determine any change over time, that is, according to age, in autistic traits in individuals with CS, we compared ADI-R scores in three age groups: children (0–9 years old during the study period), teenagers (10–18 years), and adults (> 18 years) with a nonparametric Kruskal-Wallis test. For participants > 7 years old, we compared the ADI-R scores at the time of the study to “at age 5” score with a nonparametric paired Wilcoxon test. Scores were compared between independent subgroups by an unpaired Wilcoxon test and categorical data by chi-square or Fisher’s exact tests. The correlation between quantitative variables was estimated by the Spearman coefficient correlation (rho). To identify determinants of autistic traits, we included characteristics associated with the ADI-R score on univariate analysis at *P* < .20 in a multivariate linear regression analysis. Independent variables with skewed distributions were log-transformed. The same multivariate approach was used to identify determinants of behavior disorders, with the results of the DBC-P as the dependent variable. Two-tailed *P* < .05 was considered statistically significant. We used SAS v9.4 (SAS Inst. Inc., Cary, NC) for all analyses.

## Results

### Characteristics of individuals with CS

The study included 64 individuals with CS (35 female). The mean age was 10.7 (SD 7.1) years and median age 8.1 years (range: 9 months to 30 years). All but one had clinical typical CS, one had atypical CS, and 80% had an identified *CHD7* gene mutation.

For the 63 participants for whom we could calculate a global somatic severity score, the mean score was 5.7/13 (SD 2.1, median 6, Table 2). No child had the maximum score of 13. The frequency of each item of the score ranged from 17% for “cleft lip and/or palate” to 95% for “inner ear and semicircular canals anomalies,” which is part of the standard phenotype of CS.

**Table 2:** Frequency of the 13 main lesions in participants with CHARGE syndrome (CS) to calculate the global somatic severity score (*n* = 64)

Lesions	n/n with available data (%)
Inner-ear and semi-circular canals malformation	61/64 (95)
Repetitive ear infections	37/50 (74)
Coloboma	46/61 (75.4)
Cardiac malformation	38/61 (62.3)
Bone anomalies	27/47 (57.4)
Genital anomalies and puberty delay	24/47 (51.1)
Growth retardation	25/55 (45.5)
Facial palsy	22/48 (45.8)
Kidney and urinary tract malformation	17/53 (32.1)
Choanal atresia	22/59 (37.3)
Lip and palate cleft	10/58 (17.2)
Esophageal atresia	14/48 (29.2)
Microcephaly	20/55 (36.4)

The medical severity during the first year of life score could be calculated for 62 participants: 25% had a score of 0, 31% of 1, 24% of 2, 16% of 3, and 3% of 4. In total, 58% underwent surgery during their first year of life, 49% had a gastrostomy, 26% spent at least 6 months in a hospital that year, and 18% had a tracheostomy.

Among the 52 participants with at least one brain MRI (at any age), 50% had a brain anomaly score of 1 (no brain malformation except for olfactory bulb and inner ear malformations), 20% had a score of 2, 17% a score of 3, and 13.5% a score of 4.

For the 60 participants with available visual function data, 60% had minor lesions (visual impairment score of 0), 15% moderate lesions (score of 1), and 25% severe lesions, including two with retinal detachment (score of 2).

Data were available about severity of hearing loss for 57 participants: 20% had normal hearing (score of 0), 28% mild hearing loss (score of 1), 21% moderate hearing loss (score pf 2), 5% severe hearing loss (score of 3), and 26% profound hearing loss (score of 4).

The medical characteristics of the three age groups (children, teenagers, adults) did not differ significantly (Table 3).

**Table 3:** Medical scores by age group: children, teenagers, and adults

	Children (0–9 years)	Teenagers (10–18 years)	Adults (> 18 years)	Chi2	*P*^a^
Score	(*n* = 33)	(*n* = 22)	(*n* = 9)		
Global somatic severity	5.70 (2.07)	5.60 (2.21)	6.22 (1.99)	0.62	0.7321
Medical gravity during the first year	1.10 (0.87)	1.47 (1.39)	2.00 (1.10)	4.93	0.0853
Brain anomalies	1.94 (1.06)	1.85 (1.14)	2.13 (1.36)	0.21	0.9015
Hearing loss	1.88 (1.48)	2.16 (1.61)	1.64 (1.36)	0.65	0.7235
Visual impairment	0.63 (0.89)	0.60 (0.82)	0.89 (0.93)	0.83	0.6596

Data are mean (SD)

^a^Kruskal-Wallis test

### Adaptive functioning

**Fig. 1:**
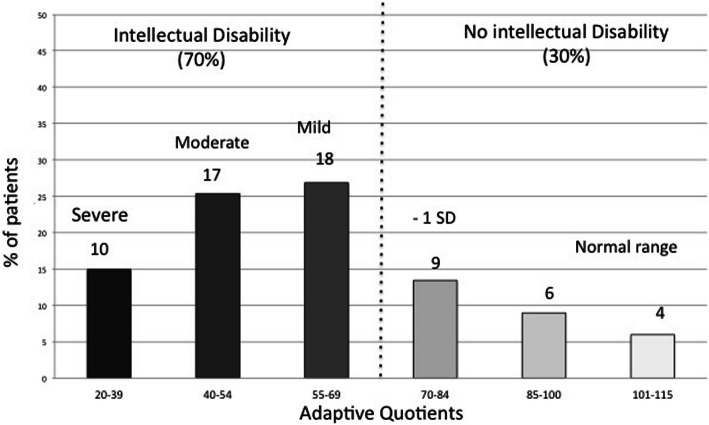
Scores for the Vineland adaptive behavior scale

**Fig. 2:**
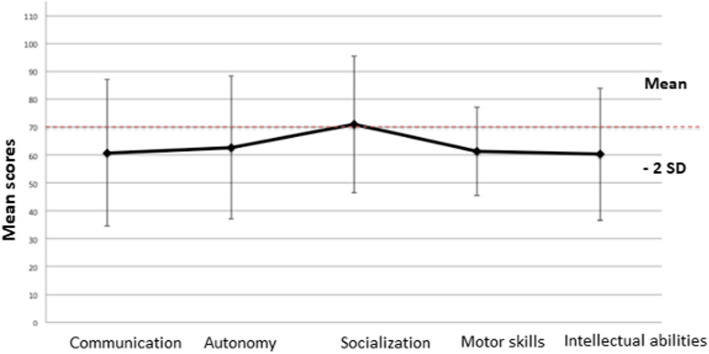
Subscores for the Vineland adaptive behavioral scale

The mean global VABS-II scores for the 64 participants was 60.3 (SD 23.6; median 57, range 20–110). Overall, 45/64 (70%) participants had impaired adaptive functioning (i.e., total score < 70, mostly mild or moderate). Only 10 (16%) had severely impaired adaptive functioning (score < 40). Of those without, only four had a score > 100 (Fig. 1). The socialization domain had the highest scores, with a mean of 71 (SD 24.5); this showed socialization capacity in the low normal range. For all other subdomains, the mean was about 60, with large SDs (Fig. 2).

### Sensory particularities

Data for the Dunn questionnaire were available for 48 participants (mean age 11 years and 11 months; SD 7 years and 2 months; range 1–30 years). Most (72%) had more particularities related to emotions and endurance than normal; 65% had sensory hyporeactivity. Half (51.3%) were more active and more restless than normal. They had poorer ability to control their emotions, to move effectively, and use their bodily sensations to manage their emotions than the normal population did (− 1 SD). No score reached − 2 SD. Despite eye and ear lesions, participants with CS could process visual, auditory, proprioceptive, and balance information and had fine motor abilities in the low range of normal.

### Frequency of ASD according to the ADI-R and DSM-5

Complete results for the ADI-R were available for 46 participants (27 females; mean age = 11.8 years, median age 7 years, and range 2–30 years). Nine children were aged 5 years or younger when their parents were interviewed for the ADI-R; these parents described only the children’s current symptoms. For the 32 patients who were 8 years or older, parents responded about both the current (interview) period and “when the child was 5”. In these results, “at age 5” refers to both the current time for the youngest children and “when they were 5” for the older patients.

Of these 46 patients, 39 (85%) had a mutation in the *CHD7* gene, and 1 in the *EFTUD2* gene. The other six had no identified mutation at the conclusion of the study.

**Fig. 3:**
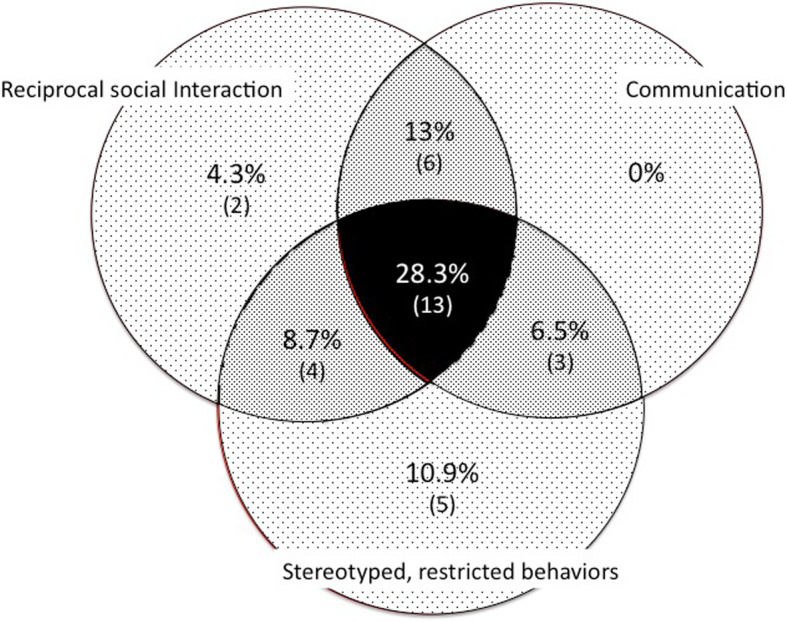
Distribution of the 33 participants whose scores exceeded the cutoff in one of the three domains of the Autism Diagnostic Interview Revised (ADI-R)

**Fig. 4:**
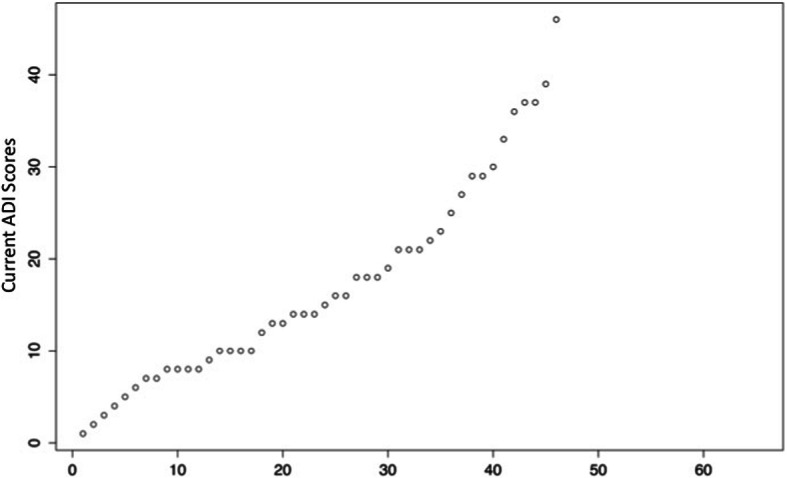
Global ADI-R scores classified from lowest to highest for the 46 participants

“At age 5”, 28% (*n* = 13, 6 females) had scores exceeding the cutoffs in all three domains and therefore might have a diagnosis of autism. The same proportion of individuals (*n* = 13, 7 females) had no score that reach any of the 3 cutoffs. Finally, the other 20 patients (43%,14 females) had scores that exceeded the cutoffs in one or two domains (*n* = 7 and *n* = 13, respectively) (Fig. 3, Table 4). Many participants (*n* = 33, 72%) had at least one score above the cutoff in one of the three domains; all exhibited at least one autistic behavior. The three domains were affected similarly. The distribution of the total ADI-R scores for the 46 participants was continuous (Fig. 4). The male/female ratio in the group of patients with autism was similar to that in the group without it. The ADI-R scores for the current period were similar, on average, to those “at age 5” (data not shown).

**Table 4:** Autism Diagnostic Interview Revised scores (at age 5) for 46 participants

Domain	Mean	SD	Range	Maximum
Social interaction	10.33	7.29	0–26	30
Nonverbal skills	1.46	1.75	0–6	6
Peer relationships	3.48	2.99	0–8	8
Failure to share pleasure	2.37	2.19	0–6	6
Social reciprocity	3.04	2.13	0–8	10
Communication				
Verbal participants (*n* = 33)	8	4.86	0–20	26
Nonverbal participants (*n* = 13)	6.69	4.79	0–14	14
Gestures	2.33	2.47	0–8	8
Make-believe play	2.57	2.40	0–6	6
Conversation (for verbal participants)	2.46	1.25	0–4	4
Stereotyped language (for verbal participants)	1.30	1.47	0–5	8
Restricted behaviors	3.13	2.22	0–9	12
Encompassing preoccupations	0.59	1.02	0–4	4
Compulsions rituals	1.37	1.36	0–4	4
Hand and finger motor mannerisms	0.74	0.80	0–2	2
Concern for part -objects	0.43	0.58	0–2	2

Analysis of the DMS-5 criteria according to the ADI-R responses “at age 5” showed that 25/46 individuals (54%) had a strict diagnosis of ASD, and 41/46 (89%) had positive results for at least one criteria in the 2 DMS-5 domains (Table 5). Severity was not classified as level 3 (very severe) for any child, was classified as level 2 for only three of them, and as level 1 (mild) for all others.

**Table 5:** Frequency of criterion for autism spectrum disorder for participants (*n* = 46) with complete ADI-R data (at age 5) by the two domains in the Diagnostic and Statistical Manual of Mental Disorders, 5th Edition (DSM-5)

DSM-5	Criterion	n (%)
A. Communication and interaction	Deficits in social-emotional reciprocity	41 (89)
	Deficits in nonverbal communicative behaviors used for social interaction	36 (78)
	Deficits in developing, maintaining, and understanding relationships Deficits in developing, maintaining, and understanding relationships	38 (83)
B. Restrictive and repetitive patterns of behaviors	Stereotyped or repetitive motor movements, use of objects, or speech	34 (74)
	Insistence on sameness, inflexible adherence to routines, or ritualized patterns or verbal nonverbal behavior	26 (56)
	Highly restricted, fixated interests that are abnormal in intensity or focus	8 (17)
	Hyper- or hyporeactivity to sensory input or unusual interests in sensory aspects of the environment	14 (30)

### Global responses to the ADI-R domains and subdomains by ASD profiles, comparing participants with CS and children with autism and with intellectual disability

The social interaction domain of the ADI-R caused fewer difficulties for participants with CS than for children with autism (mean 10.33, SD 7.29 vs 24.96, SD 4.42) but more difficulties than for children with intellectual disability (mean 3.83, SD 4.57). For the communication domain, participants with CS who were verbal were similar to children with autism (mean 8, SD 4.86 vs 10.16, SD 10.79), but had fewer difficulties than children with intellectual disability (mean 2.13 SD 3.48). Nonverbal participants with CS had significantly fewer communication difficulties than nonverbal children with autism (mean 6.69, SD 4.79 vs 12.48, SD 2.16), and communication difficulties similar to those of nonverbal children with intellectual disability (mean 2.13, SD 2.67). For the domain of restricted, repetitive behaviors, participants with CS had significantly fewer abnormal behaviors than children with autism (mean 3.13, SD 2.22 vs 6.16, SD 3.21) but more than children with intellectual disability (mean 0.61, SD 1.27).

### Subdomains of the social interaction domain (Fig. 5)

Participants with CS had significantly fewer problems using nonverbal skills to regulate social interactions (direct gaze, more social smiling, varied and appropriate facial expressions) than children with autism, and levels of problems similar to those of children with intellectual disability. They had fewer difficulties in peer relationships (more interest in other children) than children with autism but more than the children with intellectual disability. Participants with CS had fewer difficulties than children with autism in sharing pleasure and were better able to show social-emotional reciprocity (less abnormal use of the other’s body, better at offering reassurance, more appropriate facial expressions, better ability to respond to unknown adults) but less able than the children with intellectual disability.

**Fig. 5:**
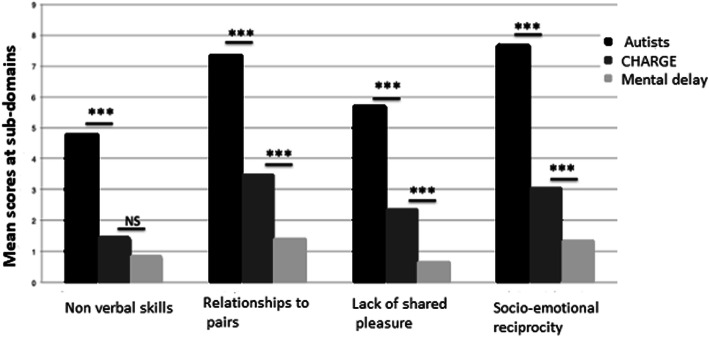
Subdomains of the “social interaction” domain between participants with CHARGE syndrome (our series) and individuals with autism and with intellectual disability (from the ADI-R manual). *** Significant difference

### Subdomains of the communication domain (Fig. 6)

Compared with children with autism, participants with CS better compensated for their language deficit by their nonverbal skills (pointing, nodding to say yes, conventional gestures). Their make-believe play was more imaginative (spontaneous imitation of an action integrated in the game, imaginative games, and imitative social games by alternating roles and initiating action). On the other hand, compared with children with intellectual disability, those with CS had more difficulties spontaneously imitating actions in the game, playing social games by alternating roles and initiating action, and conversing; they also used more neologisms. These two groups were similar in pointing to express interest, using conventional and instrumental gestures, and playing make-believe. They used a similar level of stereotyped sentences, asked a similar level of inappropriate questions, and made similar pronoun inversions.

**Fig. 6:**
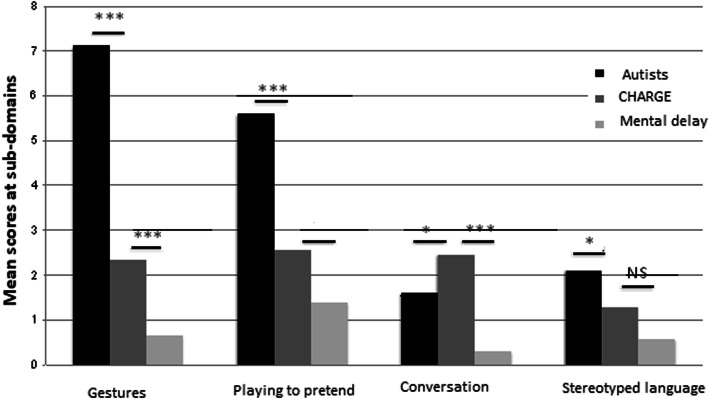
Subdomains of the “communication” domain between participants with CHARGE syndrome (our series) and individuals with autism and with intellectual disability (from the ADI-R manual)

### Subdomains of the restricted, repetitive behaviors domain (Fig. 7)

The profile of the restricted, repetitive behaviors of participants with CS differed from those of children with autism; the CS group had fewer unusual preoccupations, fewer hand, finger, and other mannerisms, and fewer preoccupations with “part-objects”, but as many compulsions and rituals. Overall, participants with CS were also similar to children with autism in difficulties in initiating activity and having conversations, and had as many rituals. They did, however, have more interest in other children and were better able to play make-believe, offer reassurance, respond favorably to another’s approach, pay attention to the other, and use the other’s body.

**Fig. 7:**
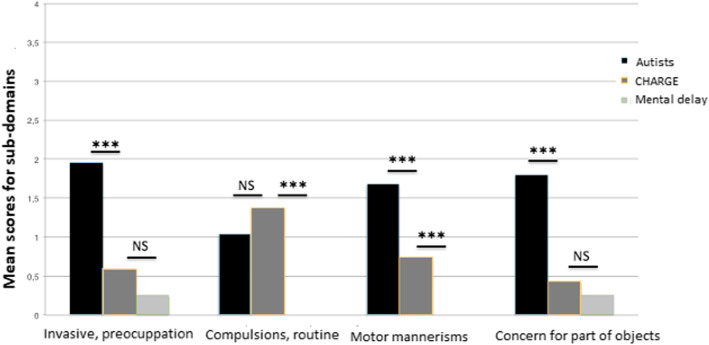
Subdomains of the “restricted behaviors” domain between participants with CHARGE syndrome (our series) and individuals with autism and with intellectual disability (from the ADI-R manual)

### Autistic traits and age

In this series, the 21 children had on average fewer autistic traits, according to ADI-R scores than either the 16 teenagers or the 9 adults (mean global score 10, SD 6 for children; 23, SD 11 for teenagers; and 26, SD 11 for adults; *P*_Kruskal-Wallis_ = .0005). The number of autistic traits changed from “at age 5” to the current age for all 32 participants who were older than 8 years at the study date: 21 (65%) had fewer autistic traits (lower ADI-R score) than at age 5, and 11 had more (higher ADI-R score). The two groups with ADI-R scores that moved in opposite directions did not differ in their adaptive functioning level, sensory particularities, or medical severity.

### Potential determinants of autistic traits

In this series, we did not find any significant correlations between ADI-R scores and the global somatic severity score, medical severity score during the first year of life, visual deficit, auditory deficit, or brain anomaly score*.* Similarly, we found no statistically significant differences between these five potential risk factors for the 13 participants who had ADI-R scores exceeding the cutoff for the three domains, and the 13 participants who had not (ADI-R scores below the cutoff for all three domains). The prevalence of the pathogenic *CHD7* variant (85%) and the ratio of truncating versus non-truncating *CHD7* mutations (30%/70%) were similar in the 13 individuals with CS and autism (ADI-R), the 13 patients without autism, and the entire group of 46 patients (data not shown). However, adaptive functioning level (VABS-II) was significantly negatively correlated with autistic traits (ADI-R score) (rho = − 0.62, *P* < .0001) (Table 6). Age of independent walking was not correlated with the ADI-R score (rho = 0.20; *P* = .2373). Consistently with this finding, ADI-R scores were lower but not significantly so for children who walked independently before rather than after age 30 months (mean 12.9 vs 20.8; *P* = .0633). Sensory particularities and autistic traits were not correlated (rho = 0.29; *P* = .07).

**Table 6:** Potential determinants of autistic traits (ADI-R score): Spearman correlation coefficient (rho)

Risk factors and determinants	rho	*P*
Score of global somatic severity	- 0.05	0.7492
Score of medical gravity during the first year	0.09	0.5351
Brain anomaly score	- 0.06	0.6969
Hearing loss score	0.09	0.5968
Visual impairment score	0.09	0.5517
Age of independent walking	0.20	0.2373
Adaptive functioning level (VABS-II)	−0.62	**0.0001**
Sensory particularities (Dunn)	- 0.29	0.07

### Behavioral disorders according to the DBC-P

**Fig. 8:**
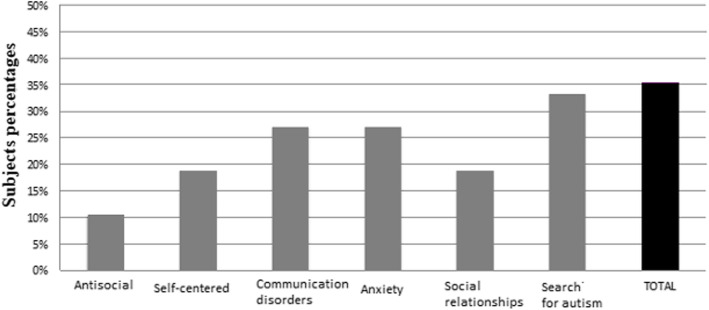
Proportion of participants whose scores exceeded the 80th percentile in each domain of the Developmental Behavior Checklist–Parents

A total of 48 participants had complete data for the DBC-P (mean age 11 years and 7 months, SD 7 years and 3 months, range 13 months to 30 years). For 21 (45%), the total score was below the cutoff, 10 (20%) had mild behavioral disorders, and 17 (35%) major behavioral disorders. Figure 8 shows the proportion of participants with scores exceeding the 80th percentile in each of the DBC-P domains.

### Potential determinants of behavioral disorders

We found a significant positive correlation between DBC-P scores and the brain anomaly score (rho = 0.44; *P* = .0068), late age of independent walking (rho = 0.44; *P* = .0059), sensory particularities (rho = − 0.48; *P* = .0011) and adaptive functioning impairment (rho = − 0.47; *P* = .0011). However, global somatic severity, medical severity during the first year, hearing loss, and visual impairment were not risk factors for behavioral disorders (Table 7).

**Table 7:** Potential determinants of behavioral disorders (Developmental Behavior Checklist-Parents score): Spearman correlation coefficient (rho)

Risk factors and determinants	rho	*P*
Score of global somatic severity	0.13	0.4059
Score of medical gravity during the first year	- 0.16	0.2836
Brain anomalies score	0.44	**0.0068**
Hearing loss score	0.06	0.6886
Visual impairment score	0.21	0.1681
Age of independent walking	0.44	**0.0059**
Adaptive functioning level (VABS-II)	−0.47	**0.0011**
Sensory particularities (Dunn)	−0.48	**0.0011**

### Correlation between adaptive functioning level and physical characteristics

Adaptive functioning level according to the VABS-II was correlated with a low brain anomaly score (rho = − 0.32; *P* = .02) and walking independently (*r* = − 0.43; = .0023). It was not correlated with global somatic severity, medical severity during the first year, or hearing or visual deficit scores. The 35 participants with a visual severity score of 0 had better adaptive functioning, on average, than the 24 participants with a visual deficit, but this difference was not significant.

### Association of autistic traits, behavioral disorders, adaptive functioning level, and sensory particularities

We found the expected significant correlation between behavioral disorders (according to the DBC-P scores) and autistic traits assessed according to the ADI-R scores (rho = 0.51; *P* = .0014). Sensory particularities were correlated with behavioral disorders (rho = − 0.65; *P* = .002), a high global somatic severity score (rho = − 0.48; *P* = .0011), a high brain anomaly score (rho = − 0.48; *P* = .0038), and adaptive functioning impairment (rho = 0.32; *P* = .0310). Sensory avoidance behaviors were correlated with severe autistic traits (rho = − 0.36; *P* = .0391), especially communication difficulties (rho = − 0.42; *P* = .0143).

On multivariate analysis, age and adaptive functioning level were independent predictors of autistic traits (*P* = .0085 and *P* < .0001, respectively). Among the variables associated with behavioral disorders on univariate analysis (brain anomalies, adaptive functioning, sensory particularities, age of independent walking, and visual impairment), both sensory particularities and adaptive functioning remained significantly associated on multivariate analysis after adjustment (*P* = .0142 and *P* = .0100, respectively).

## Discussion

We show that autism spectrum disorder, evaluated with objective standardized tools, is frequently part of the clinical phenotype of CS. This feature, like many others, is a continuum among affected individuals, ranging from very low intensity (but never null) to the cutoff of autism diagnosis for 28% of participants according to the ADI-R and 54% according to DMS-5 criteria. These two evaluations differ because of their different methods for defining autism. The ADI-R evaluates the intensity of autistic traits in three dimensions and considers a diagnosis positive of autism when the intensity of the symptoms exceeds a cutoff score in all three. The DMS-5 screens for criteria for Autism Spectrum Disorder and considers a diagnosis positive if 5 out of 7 are present in 2 dimensions, regardless of the intensity of traits, then adds a level of severity, resulting in a broader definition. In CS, autistic traits are frequent, but severe autism is not.

Our results confirm those of Hartshorne et al. [18], who showed that 27.5% of 160 individuals with CS could be considered autistic according to the Autism Behavior Checklist. Johansson et al. [21] concluded that both autism and autistic traits were present for 68% of 25 individuals with CS according to the ADI-R and the DSM-IV criteria. Again, these two published rates differ because of the tools used for analysis and the cutoffs used, but their meaning is similar. Hence, we must consider that autistic traits and autism are part of the clinical phenotype of CS.

The description of these autistic traits showed that any of the three ADI-R domains (social interaction, communication, and restricted, repetitive behaviors) can be affected as much as either of the others. Nevertheless, the subdomain analysis showed that individuals with CS could more easily develop relationships, share pleasure with him/her, and play make-believe games than children with autism. As well, individuals with CS had fewer mannerisms than children with autism. This observation may explain why the diagnosis of ASD is less often considered in children with CS. In addition, the intensity of the symptoms may be mild for most individuals with CS.

An important finding in this large series of individuals with typical CS is that that no risk factor was a significant predictor of ASD. Indeed, we found no statistically significant correlation between ADI-R scores and the participants’ physical characteristics or medical histories. ASD may thus be considered a potential feature for any child born with CS, regardless of their medical history, medical situation, or sensory deficits. This fact is important for screening and managing these symptoms, which may appear in early childhood, even in children with few sensory deficits and moderate medical conditions.

This result differs from those of Hartshorne et al. [18], most likely because these authors analyzed autistic behaviors according to non-standardized parental questionnaires and combined ASD with other challenging behaviors. The study whose design is closest to ours is that of Johansson et al. [21], who showed that autistic traits were correlated with brain malformations and intellectual disability. We confirmed the correlation between autistic traits and adaptive functioning impairment. However, waiting until the child reaches an age when delay and behavioral problems are clear may be detrimental to detecting and treating ASD features.

In this study, we differentiated ASD from behavioral disorders. Most individuals with CS have behavioral disorders or other challenging behaviors, with specificities that can mimic or differ from ASD, such as anxiety, antisocial behavior, self-injurious behavior, anger, restlessness, etc. We found these behavioral disorders linked to adaptive functioning impairment, late age of independent walking, high radiological brain malformation score, and degree of sensory particularities. We also confirmed that the level of adaptive functioning was negatively correlated with brain anomalies, age of independent walking, and visual deficiency (although not significantly) [34].

On multivariate analysis, only age and adaptive functioning level were significant predictors of autistic traits. Its cross-sectional rather than longitudinal design means that this study cannot confirm age as a determinant for ASD in CS. This question of possible pejorative evolution of ASD with age in CS, especially during adolescence, is still raised and needs further analysis. We have previously reported such a course in a girl [35]. Our multivariate analysis showed that among the severity of brain anomalies, adaptive functioning level, age of walking, and sensory particularities, only adaptive functioning level and intensity of sensory particularities explain behavioral disorders.

One major question regarding challenging behaviors in CS is whether they result from (perhaps by compensating for) a complex situation including severe medical problems, early deleterious experiences, and multisensory deficits (secondary cause) or if they have an independent origin (primary cause). According to both our results and the literature, the two mechanisms may coexist. Autistic traits may exist independent of any potential risk factors, which nonetheless remain relevant for the emergence of behavioral disorders. In this multimodal approach, we did not analyze participants’ degree of difficulties in regulating their emotions, or the quality of their multisensory integration. These dimensions, certainly crucial in the experience of people with CS [36, 37], are not easy to quantify with validated scales. However, parents, caregivers, and expert professionals know that behavioral disorders worsen in individuals with CS when they are stressed, sad, excited, or under excessive or demanding stimulation.

Including ASD in the clinical diagnostic criteria of CS for the initial diagnosis does not appear useful, because behavioral symptoms appear only over the life course.. Nevertheless, these symptoms being frequent, they should be screened to try to reduce their intensity and impact with appropriate care. All behavioral issues should not be considered only as the consequences of medical problems and sensory deficits.

## Conclusion

Children born with CHARGE syndrome, especially those with intellectual disabilities are at risk of ASD, which has to be evaluated and treated early. Children with CS with brain malformations responsible for late independent walking and with severe visual deficits are at risk of adaptive functioning impairment. If they also have sensory particularities, they are at high risk of behavioral disorders. Parents, medical, and education teams must consider behavior questions as early as possible, to try to reduce stress and stimulation that are tiring, excessive, or demanding. They must enhance all methods of communication with children with CS. The participation of pediatric psychiatrists in the care of children with CS is important, even from the first months of life.

## Data Availability

All the data are available at the Research Department (URC: Unité de Recherche Clinique) of Poitiers University, which shared the sponsorship of this study with Assistance Publique-Hôpitaux de Paris.

## Abbreviations

CS: CHARGE syndrome
ASD: Autism spectrum disorder
ADI-R: autism diagnosis interview-revised
DSM-4: Diagnostic and Statistical Manual of Mental Disorders- 4th edition
DSM-5: Diagnostic and Statistical Manual of Mental Disorders- 5th edition
DBC-P: Developmental Behavior Checklist–Parents
VABS: Vineland Adaptive Behavior Scale
